# Preliminary functional analysis of the subgingival microbiota of cats with periodontitis and feline chronic gingivostomatitis

**DOI:** 10.1038/s41598-021-86466-x

**Published:** 2021-03-25

**Authors:** Marjory Xavier Rodrigues, Nadine Fiani, Rodrigo Carvalho Bicalho, Santiago Peralta

**Affiliations:** 1 Department of Population Medicine and Diagnostic Sciences, Cornell University, Ithaca, NY 14853 USA; 2 Department of Clinical Sciences, Cornell University, Ithaca, NY 14853 USA

**Keywords:** Periodontitis, Microbial genetics, Dental diseases

## Abstract

The subgingival microbial communities of domestic cats remain incompletely characterized and it is unknown whether their functional profiles are associated with disease. In this study, we used a shotgun metagenomic approach to explore the functional potential of subgingival microbial communities in client-owned cats, comparing findings between periodontally healthy cats and cats with naturally occurring chronic periodontitis, aggressive periodontitis, and feline chronic gingivostomatitis. Subgingival samples were subjected to shotgun sequencing and the metagenomic datasets were analyzed using the MG-RAST metagenomic analysis server and STAMP v2.1.3 (Statistical Analysis of Metagenomic Profiles) software. The microbial composition was also described to better understand the predicted features of the communities. The Respiration category in the level 1 Subsystems database varied significantly among groups. In this category, the abundance of V-Type ATP-synthase and Biogenesis of cytochrome c oxidases were significantly enriched in the diseased and in the healthy groups, respectively. Both features have been previously described in periodontal studies in people and are in consonance with the microbial composition of feline subgingival sites. In addition, the narH (nitrate reductase) gene frequency, identified using the KEGG Orthology database, was significantly increased in the healthy group. The results of this study provide preliminary functional insights of the microbial communities associated with periodontitis in domestic cats and suggest that the ATP-synthase and nitrate-nitrite-NO pathways may represent appropriate targets for the treatment of this common disease.

## Introduction

Periodontal disease is a multifactorial inflammatory disease that naturally occurs in domestic cats and other mammalian species, including humans^1–4^. In general, the etiology of periodontal disease involves complex interactions between the microbial communities present in subgingival plaque and the host’s immunological response^5–7^.

Periodontal disease is clinically relevant because of its local and systemic effects and its high incidence^4,8^. The disease is characterized by local tissue inflammation and, in many cases, gradual destruction of the attachment apparatus of teeth (i.e. gingiva, periodontal ligament, alveolar bone and cementum)^1,9,10^. Two stages of disease are generally recognized: gingivitis and periodontitis^2,10^. Gingivitis is the initial and purely inflammatory stage and is considered reversible if the causative factors are controlled; periodontitis represents a more advanced and irreversible stage of the disease and is marked by loss of periodontal attachment^2^. Additionally, multiple systemic diseases and conditions have been associated with periodontal disease in people including diabetes, cardiovascular disease, and cognitive dysfunction^7,11^.

Like in people, two clinical forms of periodontitis are recognized in cats: chronic periodontitis (CP), characterized by slow and often silent progression until reaching an advanced stage in relatively older animals, and aggressive periodontitis (AP), characterized by earlier onset and much faster progression^2,8,9,12^. The reported prevalence of periodontal disease in cats is as high as 98% with no apparent sex or breed predisposition^1,8^. Common clinical signs in cats include tooth mobility and tooth loss, oral pain and discomfort, difficulty prehending food, ptyalism, halitosis, and weight loss^2,13^. Extensive and severe forms of periodontitis also occur in cats with feline chronic gingivostomatitis (FCGS)^14^. Feline chronic gingivostomatitis is a poorly understood inflammatory disease of unknown etiology characterized by severe inflammation and ulceration of oral mucosal surfaces^15,16^, and it is unknown whether the disease mechanisms that result in periodontal destruction are comparable to CP and AP.

Even though the microbial and immunological pathogenesis of periodontal disease in cats has not been fully elucidated, recent molecular studies have revealed some of the microbial features of the disease^12,17–20^. For example, we previously used 16S rRNA gene sequencing technology to show that the subgingival microbial taxonomical profile of periodontally healthy cats significantly differs to that of periodontally diseased cats^12^. Additionally, we showed that taxonomical differences underlie the different clinical presentations of the disease (i.e. CP, AP and FCGS). Furthermore, when comparing the relative abundance of prevalent taxa, and alpha and beta-diversity among cohorts, our findings were consistent with the ecological plaque and dysbiosis hypotheses of disease previously described in people^21,22^, suggesting conserved mechanisms of disease. Others have shown that the microbial composition in cats change as the periodontium transitions from health to the early stages of disease^17^, underscoring that the subgingival microenvironment is closely related to disease initiation and progression.

As remarkable as microbial composition studies are, they do not offer mechanistic insights because they do not take into consideration the functional characteristics of the microbial communities involved^22^. Indeed, it has been shown that the functional characteristics of subgingival microbial communities are more important drivers of the host’s immunological response in people than their actual taxonomical composition^22,23^. Contrasting with humans, subgingival microbial functional studies in domestic cats are lacking and it is unknown whether comparable pathogenic mechanisms and underlying microbial patterns occur. Understanding the pathogenesis of periodontal disease from a microbial functional perspective is important for veterinary medical applications and may serve as a basis for future development of novel preventive, diagnostic and therapeutic interventions that can complement or replace decades-old traditional approaches that lack a rationale. Additionally, understanding the disease mechanisms in this species will be relevant from a comparative perspective and may support leveraging client-owned cats as pre-clinical models for translational research applications. Herein, we used a shotgun metagenomic approach to explore the functional potential of subgingival microbial communities in client-owned cats, comparing findings between periodontally healthy cats and cats with naturally occurring CP, AP and FCGS.

## Results

### Sequencing data

Forty subgingival samples from different client-owned cats were sequenced, with only one failing quality control by the MG-Rast server. The other 39 metagenomic samples were distributed as follows based on periodontal status as determined by standard-of-care clinical and radiographic findings: 10 independent samples from cats diagnosed with AP, 14 independent samples from cats diagnosed with CP, 9 independent samples from cats diagnosed with in the FCGS, and 6 independent samples from periodontally healthy cats. Notably a total of 55 cats were initially sampled; however, the total genomic DNA concentration obtained was insufficient for sequencing in 15 cases, many of which corresponded to periodontally healthy cats. Shotgun sequencing requires a minimum input of 1 ng DNA and a large number of samples had too low concentration (< 0.010 ng/µL). Furthermore, only one site per cat was sampled to minimize potential bias associated with site-specific microbiome variation, which may have influenced DNA concentration. Also, given that the amount of host DNA present in each sample remains undetermined during sample preparation, it is possible that the number of detected functional features was impacted. These technical limitations are reflective of some of the challenges encountered when conducting metagenome-based research using clinical samples. Given these technical limitations, the data presented here should be considered preliminary but may be used to guide future studies focused on the microbial pathogenesis of periodontal disease in cats.

A summary of sample identification and sequences data post quality control is presented in Table 1. The analyzed dataset contained 10,697,668 sequences, with a median of 285,686 sequences per sample (101,709 to 438,659 sequences per sample). The median of 167,839 (62,991 to 243,166 features per sample) processed predicted protein features and a median of 22,217 (5503 to 64,544 features per sample) identified protein features. All sequences were deposited in the MG-Rast server and more information can be found under the sample identification.

**Table 1:** General information of subgingival metagenomes from heathy cats (Healthy_) and cats with aggressive periodontitis (AP_), chronic periodontitis (CP_) and feline gingivostomatitis (FCGS_).

MG-RAST ID	Status	Metagenome name	Sequences count	Processed: predicted protein features	Alignment: identified protein features
mgm4886070.3	AP	AP_S10	167,883	95,396	8184
mgm4886097.3	AP	AP_S11	281,256	173,663	55,984
mgm4886082.3	AP	AP_S13	336,145	188,159	18,937
mgm4886096.3	AP	AP_S18	224,652	131,182	12,692
mgm4886102.3	AP	AP_S26	417,758	222,424	45,603
mgm4886090.3	AP	AP_S29	291,489	167,839	17,176
mgm4886086.3	AP	AP_S3	168,926	88,181	5503
mgm4886076.3	AP	AP_S31	135,945	82,334	16,460
mgm4886100.3	AP	AP_S6	317,386	175,027	11,106
mgm4886084.3	AP	AP_S7	266,358	173,593	57,375
mgm4886089.3	CP	CP_S14	343,852	205,083	36,999
mgm4886066.3	CP	CP_S15	438,659	243,166	22,217
mgm4886088.3	CP	CP_S17	217,441	143,244	31,853
mgm4886068.3	CP	CP_S19	314,399	198,969	29,747
mgm4886080.3	CP	CP_S2	352,458	180,493	9514
mgm4886072.3	CP	CP_S20	242,863	146,571	14,432
mgm4886071.3	CP	CP_S21	183,495	112,716	27,487
mgm4886078.3	CP	CP_S22	286,356	164,094	14,471
mgm4886098.3	CP	CP_S24	373,276	222,367	64,544
mgm4886103.3	CP	CP_S25	290,276	183,273	38,294
mgm4886087.3	CP	CP_S27	279,231	154,658	17,444
mgm4886091.3	CP	CP_S4	409,485	217,949	42,939
mgm4886069.3	CP	CP_S5	394,560	232,947	39,668
mgm4886081.3	CP	CP_S8	177,158	110,561	30,267
mgm4886085.3	FCGS	FCGS_S1	164,898	91,380	8014
mgm4886073.3	FCGS	FCGS_S12	156,083	88,129	5710
mgm4886094.3	FCGS	FCGS_S16	101,709	62,991	8057
mgm4886074.3	FCGS	FCGS_S28	265,576	144,725	7810
mgm4886101.3	FCGS	FCGS_S30	223,690	131,268	13,984
mgm4886099.3	FCGS	FCGS_S32	309,766	175,680	19,100
mgm4886095.3	FCGS	FCGS_S33	184,690	107,933	23,029
mgm4886067.3	FCGS	FCGS_S34	360,184	195,434	31,325
mgm4886083.3	FCGS	FCGS_S35	346,862	207,229	19,111
mgm4886092.3	Healthy	Healthy_S36	316,666	182,470	26,231
mgm4886104.3	Healthy	Healthy_S9	163,290	109,115	23,993
mgm4886093.3	Healthy	Healthy_S89	285,686	141,246	7851
mgm4886077.3	Healthy	Healthy_S90	281,636	145,686	46,036
mgm4886079.3	Healthy	Healthy_S91	307,067	170,345	42,177
mgm4886075.3	Healthy	Healthy_S92	318,558	168,162	45,002

### Microbial community composition

**Figure 1:**
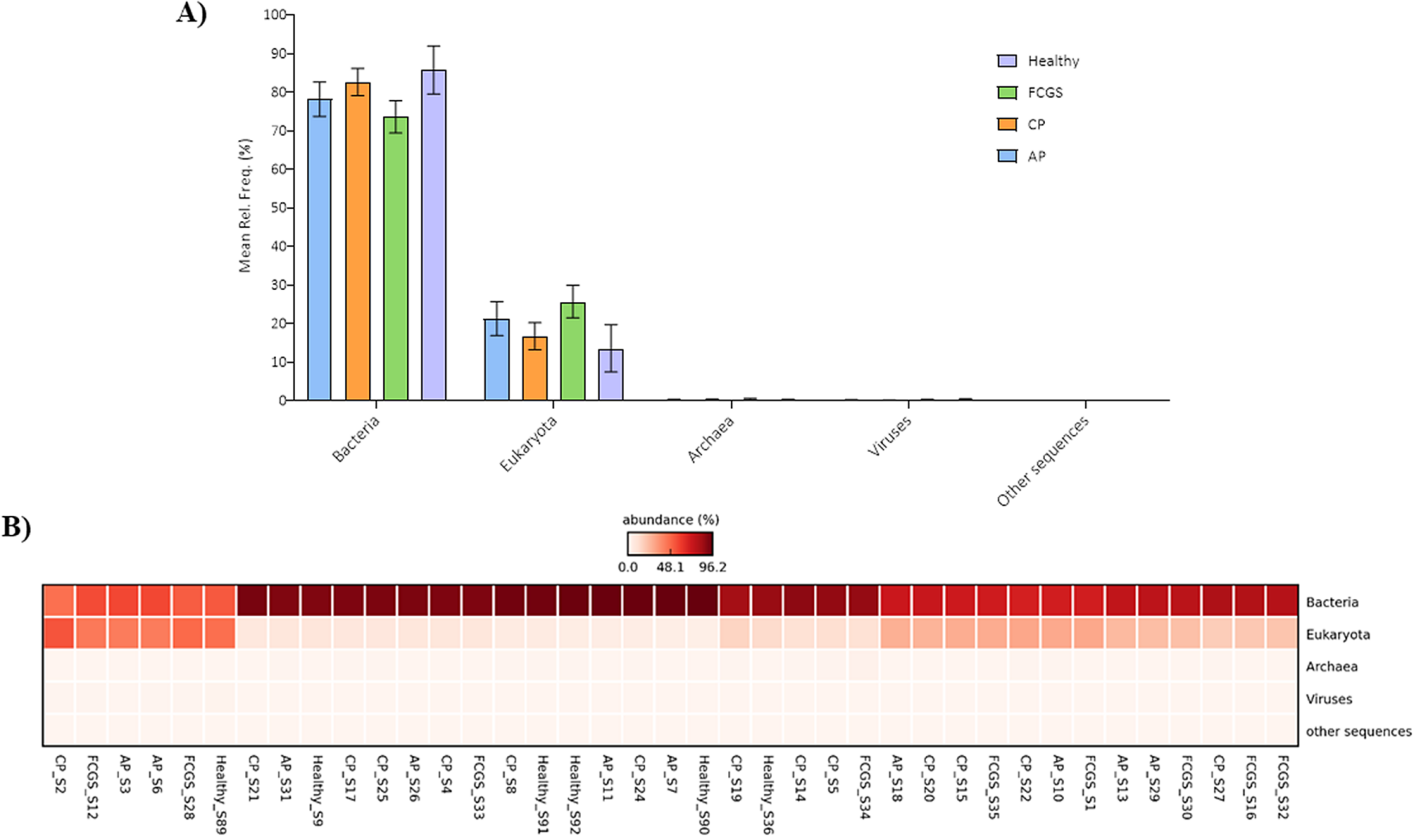
Relative abundance at domain level in subgingival metagenomes from healthy cats and cats diagnosed with aggressive periodontitis (AP_), chronic periodontitis (CP_), and feline gingivostomatitis. (**A**) Bar graph representing the relative distribution (mean relative frequency (%) and standard error of the mean) at domain level among healthy and diseased cats. (**B**) Heatmap plot illustrating abundance (%) at domain level in each metagenomic sample analyzed. (**A**) Generated using GraphPad Prism 9 (GraphPad Software LLC, La Jolla, CA, https://www.graphpad.com). (**B**) Generated using STAMP v2.1.3 (Statistical Analysis of Metagenomic Profiles) software (https://beikolab.cs.dal.ca/software/STAMP).

**Figure 2:**
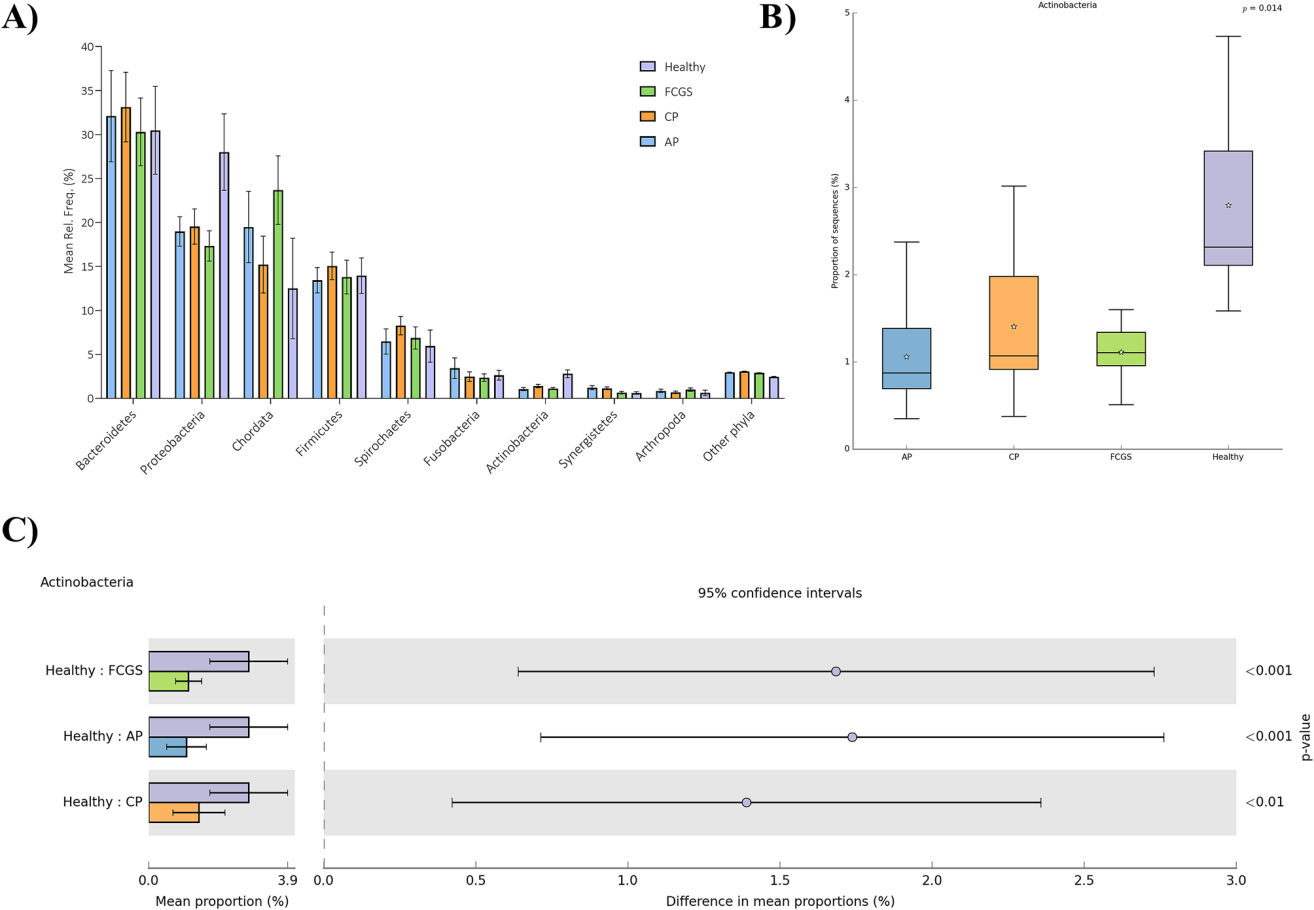
Distribution of phylum in subgingival metagenomes from healthy cats and cats diagnosed with aggressive periodontitis (AP), chronic periodontitis (CP) and feline gingivostomatitis (FCGS). (**A**) Bar graph representing the relative distribution (mean relative frequency (%) and standard error of the mean) of the most abundant phyla, minimum of 1% of abundance, in subgingival samples from cats. (**B**) Box and whiskers plots illustrating the mean (star), median (line within the box), quartiles (IQR; 75th to 25th of the data), maximum and minimum (whiskers extend to the most extreme value) proportion of sequences (%) of the phylum found with significant statistical difference in the metagenomic datasets studied (false discovery rate, FDR *P*-value < 0.05). The results were filtered using a *P*-value of 0.05 in STAMP v2.1.3 (Statistical Analysis of Metagenomic Profiles) software. (**C**) Extended error bar plots representing abundance of Actinobacteria that showed significant higher abundance in healthy cats compared to diseased cats using Tukey–Kramer with Benjamin-Hochberg FDR correction (*P*-value < 0.05). (**A**) Generated using GraphPad Prism 9 (GraphPad Software LLC, La Jolla, CA, https://www.graphpad.com). (**B**, **C**) Generated using STAMP v2.1.3 (Statistical Analysis of Metagenomic Profiles) software (https://beikolab.cs.dal.ca/software/STAMP).

**Figure 3:**
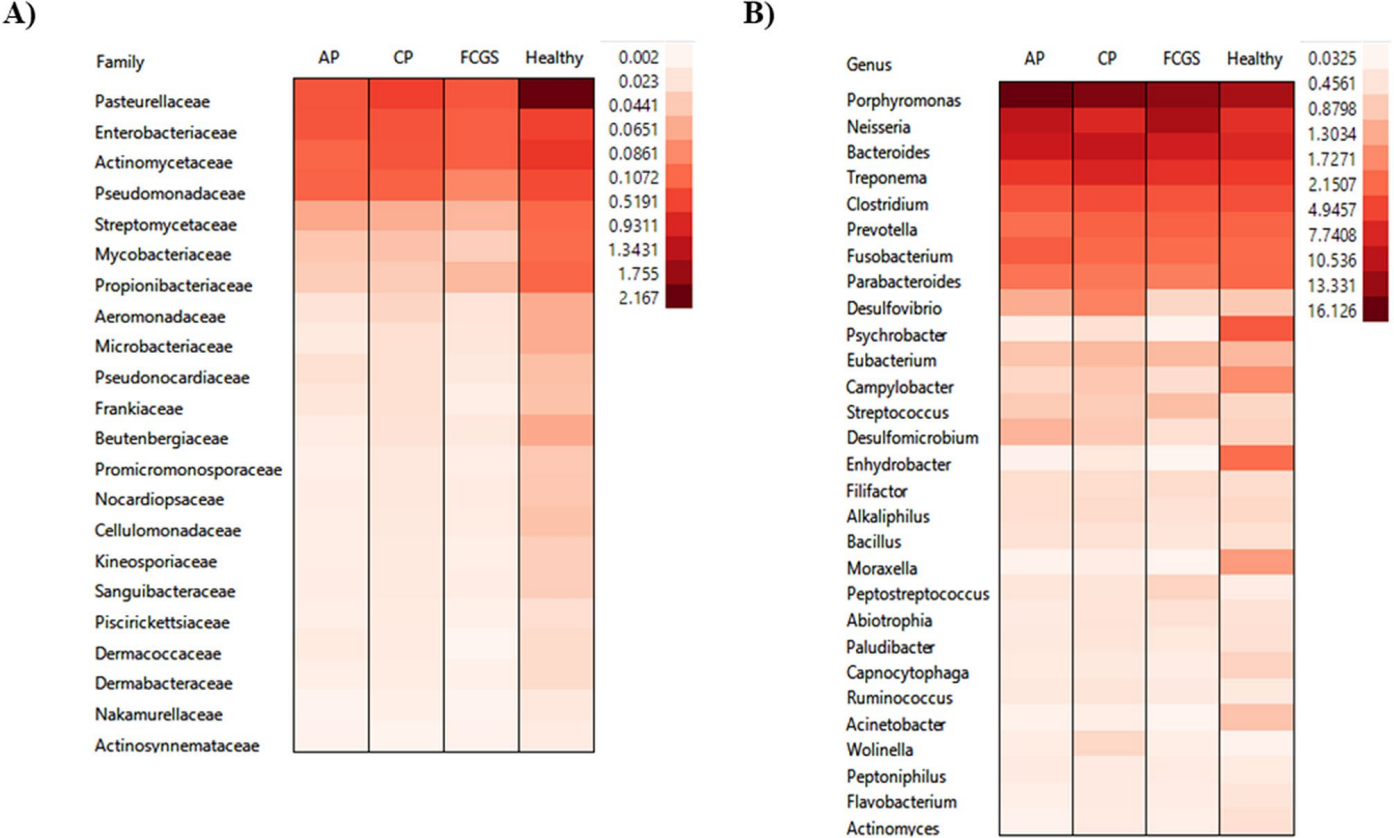
Comparison of the microbial populations in subgingival sites of healthy cats and cats diagnosed with aggressive periodontitis (AP), chronic periodontitis (CP) and feline chronic gingivostomatitis (FCGS). (**A**) Distribution of families found be statically different among groups of subgingival samples using ANOVA and Tukey–Kramer with Benjamin-Hochberg FDR correction (*P*-value < 0.05). (**B**) Overall distribution of the thirty most abundant bacterial genera identified in subgingival metagenomic samples from healthy cats and diseased cats. Cells plots were built in JMP 15 (SAS Institute Inc., Cary, NC, https://www.jmp.com/en_us/home.html) using mean relative frequency (%) calculated in STAMP v2.1.3 (Statistical Analysis of Metagenomic Profiles) software (https://beikolab.cs.dal.ca/software/STAMP).

Hits were observed in all domains with a predominance of Bacteria (Fig. 1). The most abundant phyla (minimum of 1% of abundance) are shown in Fig. 2A, including seven Bacteria taxa (Bacteriodetes, Proteobacteria, Firmicutes, Spirochaetes, Fusobacteria, Actinobacteria, and Synergistetes), and two Eukaryota taxa (Chordata and Arthropoda). These observations were unsurprising given the eating habits and hunting behavior of cats. However, we did not investigate the Eukaryota taxa further. Among Bacteria, statistical analysis showed that the relative abundance of Actinobacteria significantly differed among groups (corrected *P*-value 0.014). Subgingival samples from healthy cats (Fig. 2B) showed a significantly higher abundance of Actinobacteria (2.79% ± 1.08%) compared to cats in the CP (1.40% ± 0.7%; *P-*value < 0.01), AP (1.06% ± 0.55%; *P*-value < 0.001) and FCGS (1.11% ± 0.36%; *P*-value < 0.001) groups (Fig. 2C). Among all taxa, twenty-two families varied significantly (corrected *P*-value < 0.05) among groups (Fig. 3A). Interestingly, all these families were found to be significantly more abundant in healthy cats (*P*-value < 0.05) compared to diseased cats. The most abundant family was *Pasteurellaceae*, the proportion was higher (corrected *P*-value = 0.005) in healthy cats (2.17% ± 1.58) compared to cats with AP (0.35% ± 0.20%), CP (0.59% ± 0.40) and FCGS (0.33% ± 0.17%). In addition, the thirty most abundant bacterial genera identified in the present metagenomic datasets are shown in Fig. 3B. Among the most abundant genera, the relative abundance of *Moraxella* and *Actinomyces* were statistically different among groups; both genera were significantly more abundant (corrected *P*-value 0.019; corrected *P*-value 0.036, respectively) in healthy cats compared to diseased cats (Fig. 3B).

### Predicted functional analysis of subgingival metagenomes

#### Functional roles and gene function

**Figure 4:**
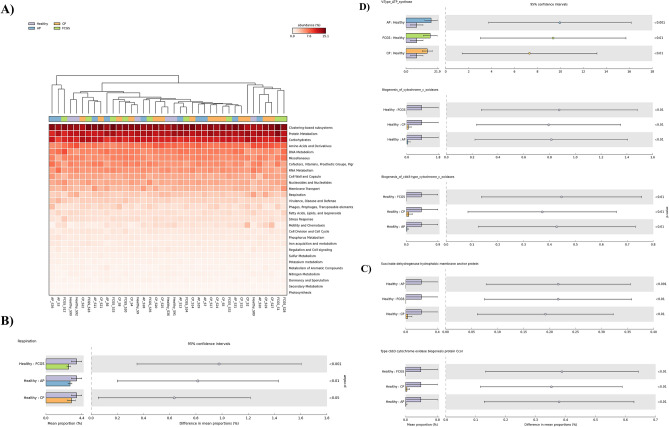
Distribution of functional annotations by SEED Subsystems of subgingival metagenomic data of healthy cats (H) and cats diagnosed with aggressive periodontitis (AP), chronic periodontitis (CP) and feline chronic gingivostomatitis (FCGS). (**A**) Heatmap dendrogram in which features at level 1 were ranked by abundance and metagenomic samples grouped by similarity (unweighted pair group method with arithmetic mean, UPGMA). (**B**) Extended error bar plot showing abundance of predicted functional category, level 1, with significant statistical difference between healthy and diseased cats. (**C**) Extended bar plot illustrating the mean proportion of predict functional features at level 3. (**D**) Extended bar plot showing the mean proportion of predict functional features at function level. The results were filtered using a *P*-value of 0.05 according to ANOVA and Tukey–Kramer with Benjamin-Hochberg FDR correction (*P*-value < 0.05). All graphs were generated using STAMP v2.1.3 (Statistical Analysis of Metagenomic Profiles) software (https://beikolab.cs.dal.ca/software/STAMP).

Considering the significance and abundance of Bacteria in the present metagenomic datasets and based on previous knowledge of periodontal biofilm composition, subsequent functional analyses using SEED Subsystems were performed filtering hits for the Bacteria domain. The abundance of functional categories at Subsystems level 1 is shown in Fig. 4A. Surprisingly, samples were not clustered together according to periodontal status, and Respiration was the only category found to be significantly different among the healthy and the diseased groups (Fig. 4B). Namely, using a *P*-value filter of 0.05, a higher relative abundance of the Respiration category was found in healthy group compared to diseased groups (Fig. 4B). We then focused on the following analysis at a deeper level in this category: at Subsystems level 3, V-Type ATP-synthase, Biogenesis of cytochrome c oxidases and Biogenesis of cbb3 type cytochrome c oxidases differed significantly among groups corrected (*P*-value equal to 0.025, 0.025 and 0.019, respectively) (Fig. 4C). Abundance of V-Type ATP-synthase was significantly higher in AP (17.43% ± 4.41%; *P*-value < 0.001), CP (14.79% ± 3.52%; *P*-value < 0.01) and FCGS (16.85% ± 4.84%; *P-*value < 0.01) compared to the healthy group (7.48% ± 4.41%). The abundance of Biogenesis of cytochrome c oxidases and Biogenesis of cbb3 type cytochrome c oxidases were higher in the healthy group (mean of 0.87% and 0.44%, respectively; *P*-value < 0.01) compared to the diseased groups (mean of 0.0–0.08% and 0.0–0.07%, respectively). At Subsystems function, Succinate dehydrogenase hydrophobic membrane anchor protein (corrected *P*-value 0.049) and Type cbb3 cytochrome oxidase biogenesis protein Ccol (corrected *P*-value 0.042) were statistically different among groups, both with significantly higher abundance in healthy cats (mean of 0.21% and 0.38%, respectively) compared to the diseased groups (mean < 0.03%) (Fig. 4D).

#### Metabolic pathway

**Figure 5:**
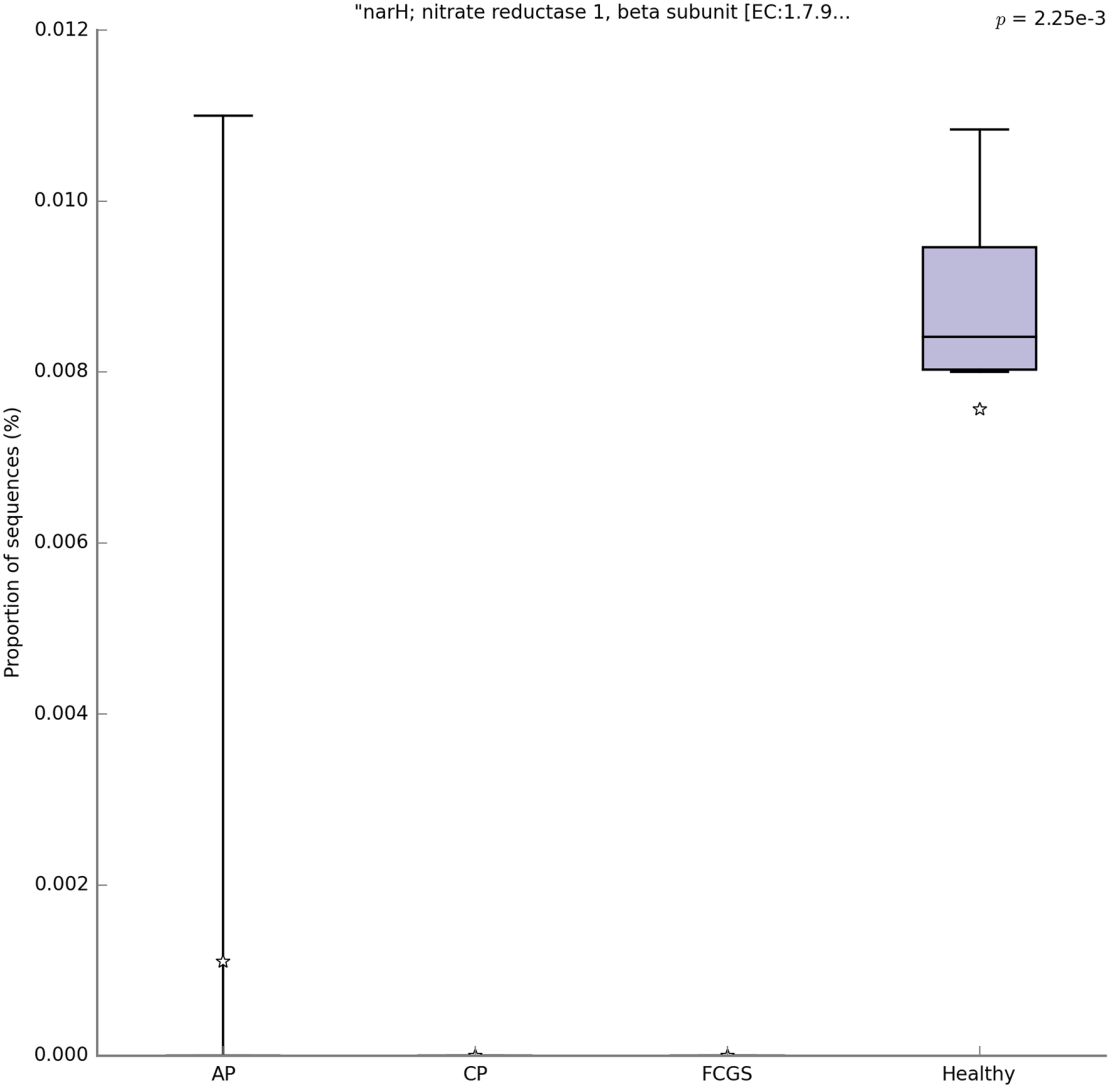
Box and whiskers plots illustrating the mean (star), median (line within the box), quartiles (IQR; 75th to 25th of the data), maximum and minimum (whiskers extend to the most extreme value) proportion of sequences (%) of the gene found with significant statistical difference in the metagenomic datasets studied (false discovery rate, FDR *P*-value < 0.05). The results were filtered using a *P*-value of 0.05; the data was computed using the MG-Rast metagenomics analysis server using KEGG Orthology (KO) database. This figure was generated using STAMP v2.1.3 (Statistical Analysis of Metagenomic Profiles) software (https://beikolab.cs.dal.ca/software/STAMP).

We analyzed genes from level 1 to function category using KEGG Orthology (KO) database and *P*-value filter for categories in STAMP v2.1.3. One gene was significantly different among groups: narH, nitrate reductase 1, beta subunit (Gene: b1225 narH; nitrate reductase A subunit beta [KO: K00371] [EC:1.7.5.1 1.7.99.-]) was found with a corrected *P*-value = 0.00225 (Fig. 5). Interestingly, this function is related to respiration and was present at a significantly higher abundance in healthy cats compared to AP, CP and FCGS (*P*-value < 0.001) (Fig. 5). The use of the KO database, molecular functions represented in functional orthologs, allows accessing the function pathway. The pathway that includes the narH gene is part of the NarL family from the Two-Component System (Pathway map: eco02020 Two-component system; class: Environmental Information Processing; Signal transduction). This was identified using the KEGG Mapper plugin for KEGG pathway mapping.

## Discussion

To our knowledge, this is the first study that uses shotgun metagenomics to investigate the compositional and functional profile of subgingival microbial communities associated with periodontal health and spontaneous periodontal disease in domestic cats. The microbiome composition reported here was consistent with previous studies and further highlights the importance of subgingival bacteria in periodontal health and disease. Importantly, significant functional features were observed in periodontally healthy and diseased cats, providing mechanistic insights of potential veterinary, comparative, and translational relevance.

Bacteria was the most abundant domain observed in the subgingival microbial communities analyzed in this study. This was expected based on bacterial colonization patterns of oral biofilms previously described in people^24–26^. The most abundant phyla were Bacteriodetes and Proteobacteria with higher abundance in the diseased and healthy groups, respectively, in accordance with previous studies^12,19^. The phylum Actinobacteria was significantly enriched in the healthy group compared to the diseased groups. This finding was consistent with a study in people which found higher proportions of Actinobacteria in subgingival communities in healthy individuals compared to those with periodontitis, in particular the genus *Actinomyces*^27^*.* Zaura et al.^28^ also identified Actinobacteria in the healthy core microbiome of oral microbial communities; and *Actinomyces*, especially, have been described as initial colonizers and compatible with periodontal health^29^. The higher relative abundance of Actinobacteria in healthy individuals might be associated with ecological succession, in which the emergence of newly dominant members occurs as biomass accumulates^27^. The presence of Actinobacteria has also been described in the oral microbiota of healthy cats^12,17,19,20,30^ and a relative abundance < 10% has been found in feline subgingival plaque samples regardless of periodontal status^17^, which is in agreement with the present study. Also, Older et al.^31^ identified lower relative abundances of Actinobacteria in FIV (feline immunodeficiency virus) positive cats with gingivitis compared to cats without gingivitis.

Unsurprisingly, the most abundant family identified in this study was *Pasteurellacea*. Indeed, unclassified *Pasteurellaceae* and *Pasteurella* spp. have previously been described as the most prevalent taxa in the oral cavity of healthy cats^20^. Some of the most abundant genera observed in this study had been previously associated with feline periodontitis and FCGS, including *Treponema*^12,17^, *Bacteroides*^18^, *Porphyromonas*^32^, *Filifactor*^12,17^, and *Peptostreptococcus*^12,17^, and with the oral and subgingival microbiota of healthy cats, including *Enhydrobacter*^12^*, Moraxella*^12,17,20^, *Capnocytophaga*^12,17,18^*.* In the present study, among the most abundant genera, the relative abundance of *Moraxella* and *Actinomyces* were significantly higher in healthy cats.

Recently, the present research group published an extensive study characterizing and comparing the subgingival microbial communities of cats affected by CP, AP, and FCGS, and healthy cats, using a 16S rRNA gene sequencing approach^12^, in which the most abundant operational taxonomic units and alpha and beta diversity were explored. Targeted 16S rRNA gene sequencing is the major tool used for bacteria and archaea identification; it is important to emphasize that the Bacteria domain was the most abundant identified in this study using whole metagenome sequencing. Whole metagenome sequencing is based on untargeted DNA sequencing of all genomes within a sample; thus, an important technical challenge is the predominance of host DNA^33^. Some samples can harbor up to 90% of host DNA, and these type of samples demand a high quantity of sequences to obtain a reasonable coverage of microbial genomes^33^.

Regarding the functional profiles, it was interesting to observe that samples from healthy cats did not cluster using the SEED database and the Bacteria domain. This fact might be related to the small sample size of healthy cats or to the quantity and quality of the sequences. Similarly, diseased cats did not cluster based on clinical phenotype (i.e. CP, AP, FCGS). In our previous study^12^, focused on microbiota composition using the same periodontal categories, with 139 samples from 44 cats, the phylogenetic beta diversity analysis showed that the microbiota of periodontally healthy cats were distinguishable from diseased cats. One possible explanation for the lack of noticeable differences among diseased groups is that the spectrum of clinical manifestations of periodontitis in cats is determined by the host’s response and not necessarily by differences in the compositional or functional characteristics of the subgingival microbial communities. Future studies in which more animals are enrolled in each category, and in which deeper sequencing depth is achieved, will help elucidate whether functional differences of microbial communities underlie the different clinical presentations of periodontitis in cats.

Respiration was found to be significantly enriched in periodontally healthy cats, and V-Type ATP synthase was significantly more abundant among the diseased groups. ATP synthase, a highly conserved enzyme, is the principal means of energy production in microorganisms^34,35^. A higher abundance of a molecule involved with energy production in the diseased groups was not surprising considering the high bacterial load and microbial biofilm formation in periodontal disease. The relationship between bacterial adhesion, which is a required first stage during biofilm formation, and ATP levels has been previously described^36^. Indeed, enhanced metabolic activity was observed when *Escherichia coli* and *Bacillus brevis* adhered onto a surface, with ATP levels increasing up to five-fold upon adhesion compared to ATP levels in planktonic cells^36^, suggesting that increased metabolic activity may help cells colonize surfaces and that decreased metabolic activity may promote cell inactivation or death^36^.

The production of ATP by bacteria can occur by substrate-level phosphorylation of fermentable carbon sources or by oxidative phosphorylation through the respiratory chain and ATP synthase^37^. ATP synthase molecules are membrane-bound transporters that have evolved for different cells^34^. However, synthesis and hydrolysis of ATP is the only function of all forms of ATP synthases^34^. Importantly, drug-resistant mutations have been mapped to the ATP synthase enzyme^37^ and studies have revealed that ATP synthase is an appropriate target for antimicrobials^34,35,37^. Moreover, Schulz et al.^38^ characterized a type of Na^+^-driven ATP synthase from the opportunistic human pathogen *Fusobacterium nucleatum*, which is also noticeably present in dental plaque biofilms. They discovered that the c-ring of the *F. nucleatum* ATP synthase is Na^+^-coupled and its structure is different from mitochondrial c-rings, which shows an opportunity for new drug discovery against this pathogen^38^. Thus, targeting energy metabolic pathways may represent a promising approach for the development of novel antibacterial substances^37^, and might be of interest for the treatment of periodontal disease in cats.

Biogenesis of cytochrome c oxidases, biogenesis of cbb3 type cytochrome c oxidases, succinate dehydrogenase hydrophobic membrane anchor protein, and type cbb3 cytochrome oxidase biogenesis protein Ccol were all significantly enriched in the periodontally healthy group described in this study. In agreement with these findings, the predicted microbial feature aerobic respiration I (cytochrome c) was previously identified as significant for cats without gingivitis compared to cats with gingivitis using PICRUSt2 based on the V4 region of the 16S rRNA gene^31^. The cytochrome c oxidases are terminal oxidases that accept electrons from cytochrome c and transfer in reactions to produce H_2_O^39^. Castresana et al.^40^ stated that “Cytochrome oxidase is a key enzyme in aerobic metabolism”, and more specifically, ccb3 oxidases have high affinity for oxygen and low proton-translocation efficiency^41^. The cbb3 oxidase is induced under low oxygen conditions and also in microaerophilic conditions; therefore, it is recognized as an important player in low oxygen environments^41^. Accordingly, the higher abundance of these enzymes in healthy cats is consistent with biofilm progression, which leads to a change in the microflora from aerobic to anaerobic^42^. Unsurprisingly, the majority of genera described in our previous study as significantly increased in periodontally diseased cats were gram-negative and anaerobic^12^.

Also, we identified that the abundance of the gene narH (nitrate reductase) was significantly higher in healthy cats when compared to periodontally diseased cats. This gene belongs to the NarL family from the two-component signal transduction systems, which enable cells to sense and respond to stimuli by changes in their environment or in their intracellular state (https://www.genome.jp/dbget-bin/www_bget?eco02020). The protective effect that microbial nitrate reductases might have on dental diseases that involve microbial-host interactions has been previously documented^43,44^. For example, it has been shown that the production of nitrite by commensal nitrate-reducing bacteria limits cariogenic/acidogenic bacteria growth due the antimicrobial oxides of nitrogen presence^43,44^, including nitric oxide (NO). Additionally, a previous study showed that *Actinomycetales* includes effective nitrate reducing species on the tongue of healthy individuals, specifically *Actinomyces* spp.^43,45^. This finding is consistent with the higher abundance of Actinobacteria and enrichment of the nitrate reductase gene found in the periodontally healthy group of cats described in this study. On the other hand, we did not find a correlation between the narH gene and Actinobacteria in this study; additional studies will help elucidate which groups of subgingival bacteria underlie the enrichment of nitrate reductases in periodontally healthy cats.

Importantly, the reduction of nitrate to nitrite by oral bacteria has also emerged as a key pathway in systemic NO homeostasis in mammals, and its selective antimicrobial activity prevents dietary nitrate-dependent lowering of blood pressure, inhibition of platelet aggregation and ischemic injury^46^. According to Hyde et al.^45^, the potential of the entero-salivary nitrate-nitrite-NO pathway to regulate NO bioavailability maintenance by nitrate reductase of specific bacteria may unveil new paradigms on the regulation and production of endogenous NO, which may show new targets for therapeutic interventions.

In conclusion, we have confirmed taxonomic features described in previous feline and human studies that have explored the subgingival microbiome associated with periodontal health and disease. Additionally, relevant functional features were identified and compared to published metagenome data. Importantly, we identified a higher abundance of the ATP synthase gene in diseased cats, and a higher abundance of the narH (nitrate reductase) gene in healthy cats. These two predicted functional annotations may represent important targets for the treatment of periodontal disease in cats. Although this study revealed important aspects related to the functional potential of subgingival communities associated with periodontal health and disease in cats, future functional metagenomic analyses will be required to corroborate and complement these findings, and to reveal whether the predicted functional features vary across the different clinical presentations of periodontitis in cats.

## Material and methods

### Ethics statement

The research protocol was reviewed and approved by Cornell University Institutional Animal Care and Use Committee (protocol number 2015-0117). Cat owners voluntarily agreed to enroll their cats in the present study and signed informed consent was obtained prior to experimental sampling. The methods were performed according to approved guidelines.

### Study animals and sample collection

The sampled animal population consisted of 40 client-owned domestic cats originally presented to the Dentistry and Oral Surgery Service at Cornell University’s Hospital for Animals for periodontal treatment or for treatment of FCGS. The sex distribution was 20 females and 20 males. The breed distribution was 33 domestic and 7 purebred cats (2 Bengals, 2 Siamese, 1 Manx, 1 Himalayan and 1 Persian). The median age was 5 years (range = 2 to 17 years). None of the animals had received systemic antibiotics, immunosuppressive medications, or oral antiseptics during the previous 4 weeks. None of the cats had evidence of chronic debilitating systemic disease (i.e. chronic kidney insufficiency, diabetes mellitus, endocrinopathies, etc.). Periodontal evaluation and sampling were performed by a board-certified veterinary dentist using standard-of-care periodontal probing and full-mouth intraoral radiographs while animals were under general anesthesia; general anesthesia was supervised by a board-certified veterinary anesthesiologist. Each cat was assigned to one of four groups according to periodontal status (i.e. healthy, CP, AP, FCGS) following previously described clinical and radiographic criteria^12^.

Subgingival samples were obtained after radiographic evaluation but prior to any periodontal instrumentation or disinfection, or systemic antibiotic administration. Samples were collected using the endodontic paper point technique, as previously described^12,47,48^. Briefly, a sterile endodontic paper point (absorbent paper points—Coarse, Meta Dental Corp., Glendale, NY) was carefully inserted into the sulcus and gently rubbed along at least half of the circumference of individual teeth. Only one tooth was sampled per enrolled cat; only canine and carnassial teeth (maxillary fourth premolar and/or mandibular first molar teeth) were sampled. The sampled tooth was selected randomly in healthy animals. In the disease groups (i.e. AP, CP and FCGS), only samples from teeth with more than 50% attachment loss based on standard clinical parameters were included for analysis; the most severely affected tooth was sampled in each cat. Samples were aseptically collected, labeled, and stored individually in 1.5 ml sterile polypropylene microcentrifuge tubes, placed on ice, transported to the laboratory within 4 h, and then frozen at − 80 °C.

### DNA extraction

For DNA extraction, we added 1.0 mL of UltraPure distilled water (DNAse and RNAse Free) (Invitrogen, Life Technologies, USA) in 1.5 mL microcentrifuge tubes containing the paper point. The tubes were then placed in a vortex (Fisher Scientific, Hampton, NH) and mixed for 10 min at maximum speed. Paper points were discarded, and the remaining liquid was centrifuged for 10 min at 13,000 rpm at room temperature. The pellet obtained was used for extracting DNA, which was performed using DNeasy PowerFood Microbial Kit (Qiagen, Hilden, Germany) following the manufacturer's instructions. DNA concentration was determined using Qubit 2.0 fluorometer (Invitrogen, Life Technologies, Singapore) and Qubit dsDNA BR assay kit (Invitrogen, Life Technologies, USA). DNA samples were normalized to 0.2 ng/µL with UltraPure distilled water (DNAse and RNAse Free) (Invitrogen, Life Technologies, USA) before DNA sequencing.

### Shotgun metagenomic sequencing

Normalized DNA samples were processed using the Nextera XT DNA Library Prep Kit (Illumina Inc., USA). The gDNA tagmentation, libraries amplification (amplification with unique combination of indexes adapters (Nextera XT Index Kit, Set A, Illumina Inc., USA)), clean up, normalization, and pool of libraries were performed following the Nextera XT DNA Library Prep Kit Reference Guide (Illumina Inc., USA). The library obtained and PhiX sequencing control v3 (Illumina Inc., USA) were diluted and denatured according to MiSeq System Denature and Dilute Libraries Guide (Illumina Inc., USA). The final library was prepared using 95% of denatured library and 5% of denatured PhiX 20 pM in a final volume of 600 µL. The combination of library and PhiX control was loaded in MiSeq Reagent Kit v3 600 cycles (Illumina Inc., USA). Lastly, the pair-end sequencing was performed through the Illumina MiSeq Plataform (Illumina Inc., USA).

### Bioinformatics and statistical analysis

The raw data files were demultiplexed based on index information (Casava v.1.8.2, Illumina Inc.). The reorganized fastq files (read 1 and read 2) were merged using “join paired-ends” in MG-Rast metagenomics analysis server v. 4.0.3.^49^; in this server the reads were also annotated and binned. In MG-Rast, sequences were submitted to data hygiene (quality control and removal of artifacts), feature extraction, feature annotation, and profile generation. The M5nr, a non-redundant database, analyzed the results against different reference databases and the number of hits distributed in these databases were displayed in the shotgun metagenomes profiles. Considering the number of annotated reads, RefSeq (NCBI Reference Sequence Database)^50^ was selected for taxonomic annotation and SEED Subsystems^51^ and KEGG^52,53^ Orthology (KO) databases for functional annotation. To better understand the functional features analyzed, a brief analysis of the microbial community of these metagenomes was performed. Maximum e-value of 1 × 10^−5^, 60% minimum identity, minimum alignment length cut-off of 15, minimum abundance of 1, and representative hit were applied for all analyses. The representative hits approach creates counts additive across functional and taxonomic levels allowing the comparison of functional and taxonomic profiles of different metagenomes. Also, the MG-Rast server the plugin-hub KEGG mapper^52^ was used to illustrate the predicted metabolic pathway in metagenomic samples.

The data obtained from the MG-Rast server was uploaded into STAMP v2.1.3 (Statistical Analysis of Metagenomic Profiles) software package^54^. Calculation of abundance of features, statistical analysis of the metagenomes, heatmap dendrograms, extended error bar plots, and box and whiskers plots were performed using STAMP v2.1.3 software. Analysis of variance (ANOVA) followed by Tukey–Kramer post hoc test and multiple test correction Benjamin- Hochberg FDR (*P*-value < 0.05) were used to compare taxonomic and functional features among groups. Features were filtered based on *P*-value to created plots using only relevant features. Heatmap dendrograms were built using mean abundance (%), average neighbor (unweighted pair group method with arithmetic mean, UPGMA) and dendrogram clustering threshold of 0.75. Cell plots and bar graphs using mean relative frequency (%) of taxa were fitted using JMP 15 (SAS Institute Inc., Cary, NC) and GraphPad Prism 9 (GraphPad Software LLC, La Jolla, CA), respectively. Unclassified reads were removed from analysis.
